# Topical Application of Oleuropein Induces Anagen Hair Growth in Telogen Mouse Skin

**DOI:** 10.1371/journal.pone.0129578

**Published:** 2015-06-10

**Authors:** Tao Tong, Nahyun Kim, Taesun Park

**Affiliations:** Department of Food and Nutrition, Brain Korea 21 PLUS Project, Yonsei University, Seoul, South Korea; University of Ulm, GERMANY

## Abstract

We observed that oleuropein, the main constituent of the leaves and unprocessed olive drupes of *Olea europaea*, protected mice from high-fat diet-induced adiposity by up-regulation of genes involved in Wnt10b-mediated signaling in adipose tissue. The activation of Wnt/β-catenin pathway is also well established to positively regulate the anagen phase of hair growth cycle in mice skin.

**Methodology and Principal Findings:**

Oleuropein promoted cultured human follicle dermal papilla cell proliferation and induced LEF1 and Cyc-D1 mRNA expression and β-catenin protein expression in dermal papilla cells. Nuclear accumulation of β-catenin in dermal papilla cells was observed after oleuropein treatment. Topical application of oleuropein (0.4 mg/mouse/day) to C57BL/6N mice accelerated the hair-growth induction and increased the size of hair follicles in telogenic mouse skin. The oleuropein-treated mouse skin showed substantial upregulation of Wnt10b, FZDR1, LRP5, LEF1, Cyc-D1, IGF-1, KGF, HGF, and VEGF mRNA expression and β-catenin protein expression.

**Conclusions and Significance:**

These results demonstrate that topical oleuroepin administration induced anagenic hair growth in telogenic C57BL/6N mouse skin. The hair-growth promoting effect of oleuropein in mice appeared to be associated with the stimulation of the Wnt10b/β-catenin signaling pathway and the upregulation of IGF-1, KGF, HGF, and VEGF gene expression in mouse skin tissue.

## Introduction

The hair follicle, a specific mini-organ appendage of the skin, is composed of epidermal (epithelial) and dermal (mesenchymal) compartments, and their interaction plays an important role in the growth of the hair follicle [1,2]. Hair follicle formation largely takes place during fetal and perinatal skin development [3]. After birth, mature and actively growing hair follicles eventually become anchored in the subcutis and are periodically regenerated by spontaneously undergoing repetitive cycles of growth (anagen), apoptosis-driven regression (catagen), and relative quiescence (telogen) [3]. In each cycle, a new hair shaft is formed, and the old hair is eventually shed, mostly in an actively regulated process termed the exogen phase [3]. The length of the hair shaft corresponds to the duration of the anagen phase [4]. Most common forms of hair loss (alopecia) are caused by aberrant hair follicle cycling, involving a decrease in the duration of the anagen phase and an increase in the percentage of hair follicles in the telogen phase [4]. Based on this knowledge, the goals for treating alopecia include converting telogen hair follicles to anagen hair follicles and prolonging the anagen phase.

Wnt signaling pathways are divided into two groups according to their signal transduction pathways: canonical Wnt signaling in which β-catenin stabilization occurs, and non-canonical Wnt signaling pathway in which Ca^2+^ flux or activation of the Jun N-terminal is involved [5]. Canonical Wnt signaling is involved in almost every aspect of embryonic development and controls homeostatic self-renewal in several adult tissues [5]. Mutations in the canonical Wnt signaling pathway destroy the homoeostatic balance in these tissues to cause pathological conditions such as cancer or disturbances in skeletal bone mass [5]. The Wnt/β-catenin pathway plays important roles in hair follicle regeneration and could be an ideal target for the development of drugs that activate hair growth by inducing anagen-phase genes [6]. Recent studies showed that β-catenin activity is present in the dermal papilla in the actively growing anagen phase and ablation of β-catenin in the dermal papilla leads to premature induction of the catagen phase in mice [6].

Oleuropein, the main constituent of the leaves and unprocessed olive drupes of *Olea europaea*, is a polyphenol belonging to the secoiridoid class [7]. This phenolic compound has several pharmacological properties, including anticancer, hypolipidemic, and skin protective effects [8–10]. A recent study reported that oleuropein markedly reduced the viability of human MCF-7 cells (breast cancer cells) as a result of increased apoptosis [8]. In addition, oleuropein has been found to significantly attenuate high-fat diet-induced elevations in hepatic concentrations of cholesterol, triglycerides, and free fatty acids in mice [9]. Oleuropein has also been shown to prevent chronic ultraviolet B-induced skin damage in mice [10]. Recently, we observed that oleuropein reduced body weight gain and visceral adiposity in high-fat diet (HFD)-fed mice. The protective effect of oleuropein against HFD-induced adiposity in mice appeared to be mediated through the upregulation of genes involved in Wnt10b-mediated signaling cascades [11]. In the present study we investigated whether oleuropein could induce anagenic hair growth in C57BL/6N mice and explored the underlying mechanism.

## Materials and Methods

### Cell culture

Human follicle dermal papilla cells (DPCs) were purchased from PromoCell (PromoCell, Heidelberg, Germany) and maintained in follicle DPC growth medium containing supplement mix (PromoCell) at 37°C in 5% CO_2_. When the cells reached 80% confluence, they were subcultured in 4-(2-hydroxyethyl)-1-piperazine ethanesulfonic acid-buffered saline solution, trypsin/ethylenediaminetetraacetic acid (EDTA) solution, and neutralizing solution (PromoCell). To investigate whether oleuropein regulation of LEF1, CycD-1, IGF-1, HGF, VEGF, and KGF occurs indirectly through newly synthesized transcription factors, the effect of the protein synthesis inhibitor, cycloheximide (CHX; Sigma-Aldrich, MO, USA) was determined. Trypsinized DPCs were allowed to attach overnight and then treated (60 min) with cycloheximide (10 μg/ml) and thereafter switched to medium with or without oleuropein (20 μM) in the presence of cycloheximide (10 μg/ml) for 24 h. Control cells were treated similarly except that no cyclohexmide was included at any time.

### MTT assay and trypan blue exclusion assay

The proliferation of DPCs was evaluated by measuring their metabolic activity using a 3-[4,5-dimethylthiazol-2-yl]-2,5-diphenyltetrazolium bromide (MTT) assay [12]. The MTT assay was performed as follows: DPCs (3 × 10^4^ cells/well) in a 96-well microplate were treated with 10, 20, and 50 μM oleuropein (No. 0204, ≥90% purity, Extrasynthese, Genay, France) or DMSO as a control. After 24 h of incubation, 0.1 mg of MTT (Sigma-Aldrich) was added to each well, and the cells were then incubated at 37°C for 4 h. The purple formazan product created by the reduction of MTT by succinyl dehydrogenase in the mitochondria of living cells was solubilized in 100 μL of an organic solvent for 5–10 min at room temperature. The plate was read at 590 nm in a plate reader (Beckman Coulter, CA, USA). Triplicate wells were used to determine the oleuropein concentration, and all of the reported experiments were performed at least three times. To corroborate the cell proliferation result derived from MTT assay, trypan blue exclusion assay were performed. At the end of oleuropein treatment, triplicate wells of viable cells for each concentration were counted on a hemacytometer after trypsinization. Each well had three repeats of counting. The experiment was repeated three times.

### Animal experiments

Male C57BL/6N mice (6 weeks old) were purchased from Orient Bio (Seongnam, Korea) and maintained under a 12-h light–dark cycle with free access to food and water. Before the experiments, all mice were fed a commercial diet for 2 weeks for acclimation. All animals were shaved using an animal clipper at 8 weeks of age, at which time all of the hair follicles were synchronized in the telogen phase. The mice were divided into three groups of eight mice each. Each mouse received 200 μL of the reagent applied topically with a plastic spatula to the shaved dorsal skin daily for 28 days. The control group received vehicle alone, the oleuropein group received vehicle containing 0.4 mg of oleuropein, and the minoxidil group received vehicle containing 3 mg of minoxidil. All reagents used for the hair growth test were dissolved in a vehicle composed of 50% (v/v) ethanol, 30% water, and 20% propylene glycol. The back skin of the mice was photographed, and the lengths of randomly plucked hairs were measured at 0, 1, 2, 3, and 4 weeks. At sacrifice, skin tissues were collected, snap-frozen in N_2_, and stored at -80°C. All animal experiments were performed in accordance with the Korean Food and Drug Administration guidelines. Protocols were reviewed and approved by the Institutional Animal Care and Use Committee of the Yonsei Laboratory Animal Research Center (YLARC) (Permit #: 2013–0105). All mice were maintained in a specific pathogen-free facility at the YLARC.

### Histological examination

Rectangular pieces (4-μm-thick) of the central dorsal skin were collected parallel to the vertebral line, fixed in 10% neutral formalin, and then stained with hematoxylin and eosin (H&E). Representative longitudinal sections obtained from six animals were analyzed, using an Olympus BX51 microscope (Olympus, NY, USA) at 40× magnification. The thickness of hair follicles was measured in H&E-stained sections at the level of the largest diameter of the hair bulbs with clearly visible dermal papilla. Quantitative determination of hair follicle area in these sections was conducted using a digital camera (TDI Digicam HQ, Seoul, Korea) and image analysis system (TOMORO Scope Eye, Seoul, Korea).

### Enzyme-linked immunosorbent assay

Dermal levels of insulin-like growth factor-1 (IGF-1) were determined in the animals by using a modification of a previously described method [13]. The skin was minced and homogenized in a polytron-type homogenizer using 2 mL of 1N acetic acid according to the manufacturer’s instructions. The tissue homogenates were centrifuged (3,000 × g, 10 min, 4°C), and the resulting supernatants were used for enzyme-linked immunosorbent assay (ELISA). The concentration of IGF-1 was measured using an ELISA kit (Diagnostic Systems Laboratories, TX, USA).

### RNA extraction and semi-quantitative RT-PCR analysis

Total RNA was isolated from the skin tissues and DPCs using TRIzol reagent (Invitrogen, CA, USA), according to the manufacturer’s instructions. Total RNA (4 μg) was reverse-transcribed using the Superscript II kit (Invitrogen) according to the manufacturer’s recommendations. The primers used for the polymerase chain reaction (PCR) are listed in Table 1. The PCR was programmed as follows: 10 min at 94°C; 30–33 cycles at 94°C for 30 s, 55°C for 30 s, and 72°C for 1 min; 10 min incubation at 72°C. The PCR products were size-fractionated on a 2% agarose gel and stained with ethidium bromide. The PCR products were separated and visualized as described above and the band intensities were quantified using Quantity One analysis software (Bio-Rad Laboratories, CA, USA). PCR protocols were optimized for each gene separately to be in a quantifiable (linear) range in our samples.

**Table 1:** Primer sequences used for RT-PCR.

Gene description	Sequences (5′→3′)	Annealing temperature (°C)	PCR product (bp)
Wingless-type MMTV integration site family, member 10b (Wnt10b)	F: TTTTGGCCACTCCTCTTCCT	61	183
	R: TCCTTTTCCAACCGAAAACC		
Dickkopf 1 (DKK1)	F: GGTGCACACCTGACCTTCTT	59	144
	R: GAGGGGAAATTGAGGAAAGC		
Low-density lipoprotein receptor-related protein 5 (LRP5)	F: AAGGGTCCACAAGGTCAAGG	55	520
	R: AGAAGCACAGATGGCTGCAC		
Frizzled receptor 1 (FZDR1)	F: ATGAACAGGCCTTTCGTTCT	55	484
	R: CCTCGTGTAGAACTTCCTCC		
Lymphoid-enhancer-binding factor (LEF1)	F: CCAGCTATTGTAACACCTCA	55	420
	R: TTCAGATGTAGGCAGCTGTC		
Cyclin D1 (Cyc-D1)	F: TGGGAAGTTTTGTTGGGTCA	55	144
	R: TCCTTGTCCAGGTAATGCCA		
Insulin-like growth factor (IGF-1)	F: TCAACAAGCCCACAGGGTAT	60	280
	R: ACTCGTGCAGAGCAAAGGAT		
Hepatocyte growth factor (HGF)	F: CGAGGCCATGGTGCTATACT	54	290
	R: ACACCAGGGTGATTGAGACC		
Vascular endothelial growth factor (VEGF)	F: TCTTCAAGCCATCCTGTGTG	60	165
	R: GCGAGTCTGTGTTTTTGCAG		
Keratocyte growth factor (KGF)	F: GACATGGATCCTGCCAACTT	54	686
	R: AATTCCAACTGCCACTGTCC		
Glyceraldehyde-3-phosphatedehydrogenase (GAPDH)	F: AGAACATCATCCCTGCATCC	60	321
	R: TCCACCACCCTGTTGCTGTA		

### Western blot analysis

Skin tissues and DPCs were homogenized at 4°C in an extraction buffer (100 mM Tris-HCl, pH 7.4, 5 mM EDTA, 50 mM NaCl, 50 mM sodium pyrophosphate, 50 mM NaF, 100 mM orthovanadate, 1% Triton X-100, 1 mM phenylmethanesulfonyl fluoride, 2 μg/mL aprotinin, 1 μg/mL pepstatin A, and 1 μg/mL leupeptin) and centrifuged at 13,000 × g for 20 min at 4°C. Protein samples were separated using 8% sodium dodecyl sulfate-polyacrylamide gel electrophoresis and transferred onto a nitrocellulose membranes (Amersham, Buckinghamshire, UK). The membranes were blocked with 5% bovine serum albumin (BSA) in Tris-buffered saline/Tween buffer (10 mM Tris-HCl pH 7.5, 150 mM NaCl, and 0.05% Tween 20), incubated with primary antibodies (diluted 1:1,000) overnight at 4°C, and washed 4 times in Tris-buffered saline containing 0.05% Tween 20. The following primary antibodies were used: β-catenin and β-actin (SC-7963 and SC-47778, Santa Cruz Biotechnology, CA, USA). After washing, the membranes were incubated with secondary goat anti-mouse antibodies (SC-2005, Santa Cruz Biotechnology) in Tris-buffered saline containing 0.05% Tween 20 for 1 h. The blots were then developed using a chemiluminescent detection system according to the manufacturer’s instructions (Amersham).

### Immunocytochemistry

DPCs were placed in a 12-well microplate at a density of 50,000 cells per well and cultured in DPC growth medium containing supplement mix (PromoCell) in the presence of oleuropein or vehicle control (DMSO). Cells were fixed in 4% paraformaldehyde containing 0.1% Triton X-100 for 15 min at room temperature. After blocking with 5% BSA in phosphate-buffered saline (PBS) for 1 h at room temperature, the cells were incubated with β-catenin antibody (1:100) (Santa Cruz Biotechnology) overnight at 4°C. The cells were washed with PBS and incubated with Rhodamine Red-X goat anti-mouse antibody (Invitrogen) for 1 h at room temperature. Cells were counterstained with 4,6-diamidino-2-phenylindole (Invitrogen) and examined using a LSM510 Meta confocal microscope (Carl Zeiss, Oberkochen, Germany).

### Immunohistochemistry

Paraformaldehyde-fixed paraffin-embedded tissues were cut into 4-μm sections. Paraffin sections were deparaffinized and rehydrated. For antigen retrieval, the slides were autoclaved in 10 mM sodium citrate buffer. Endogenous peroxidase was blocked with 0.345% H_2_O_2_ for 30 min, and sections were further blocked with 5% BSA in PBS for 30 min. The sections were incubated overnight at 4°C with the following primary antibodies: β-catenin (1:100; BD transduction laboratory, CA, USA) and IGF-1 (1:100; Abcam, MA, USA). Sections were incubated with biotinylated anti-mouse secondary antibody (1:200) (Dako, Glostrup, Denmark) for 1 h at room temperature, followed by incubation with avidin–biotin complexes (Vector Laboratories, CA, USA). Staining was performed with 3,3′-diaminobenzidine (DAB) (Vector Laboratories) as explained in the manufacturer’s protocol. Counterstaining was performed with Mayer’s hematoxylin (Muto Pure Chemicals, Tokyo, Japan). The DAB-stained preparations were visualized with a TE-2000 general optical microscope (Nikon, Tokyo, Japan).

### Statistical analysis

Statistical analyses were performed with SPSS 12.0 software (SPSS, IL, USA). Data are represented as the mean ± standard deviation (SD). The means of groups were compared using one-way analysis of variance or Student’s t test. *P* < 0.05 was considered significant.

## Results

### Effect of oleuropein on the proliferation of DPCs

**Fig 1:**
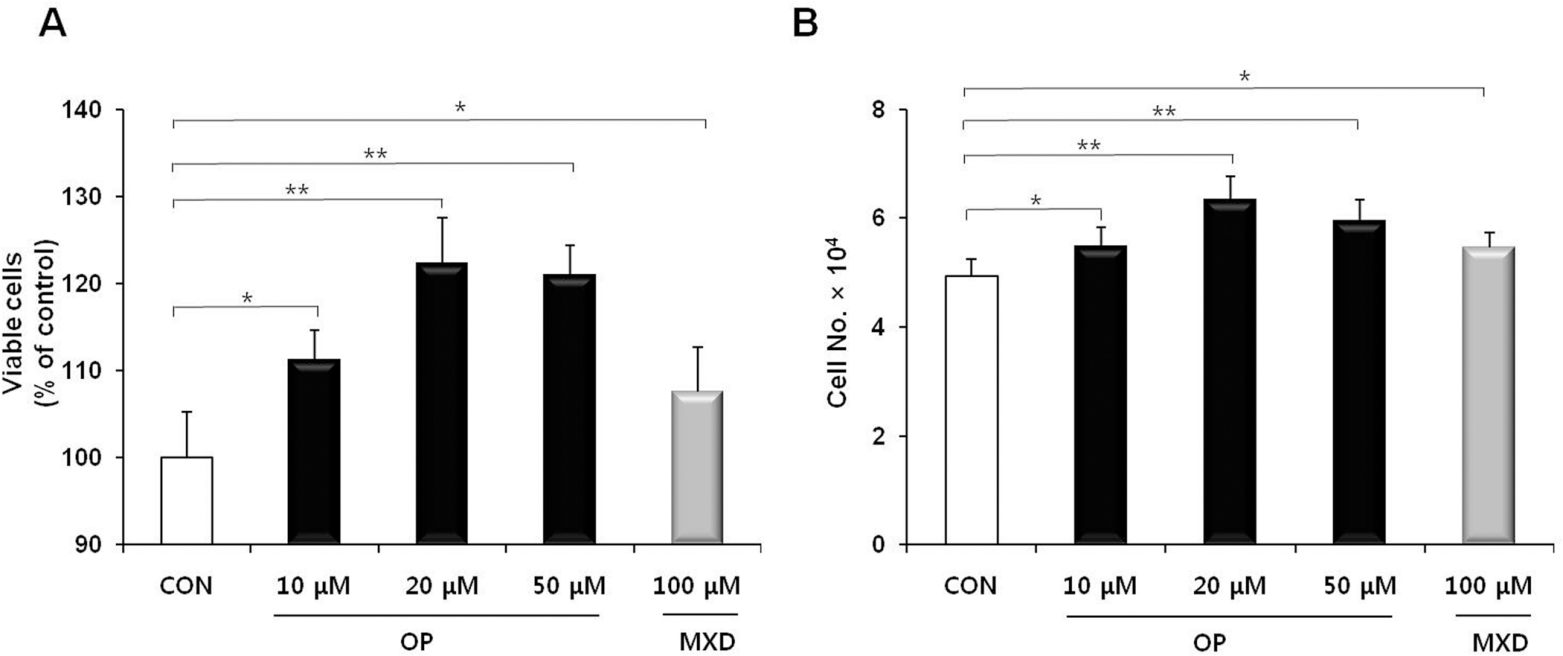
Effect of oleuropein on DPC proliferation. DPCs (3 × 10^4^ cells/well) were treated with various concentrations of oleuropein (OP) or minoxidil (MXD), as indicated. (A) Viable cells were determined by MTT assay. (B) Cells were counted by trypan blue exclusion. The values are the mean ± SD of triplicate determinations from three independent wells. * = *P* < 0.05 and ** = *P* < 0.01 *vs*. control.

We investigated the effect of oleuropein on the proliferation of DPCs. DPCs were treated with various concentrations of oleuropein (10, 20, and 50 μM), and cell proliferation after day 5 was assessed by using an MTT assay and trypan blue exclusion assay. Outcomes from trypan blue assay were highly consistent with that from MTT assay (Fig 1B). Treatment with oleuropein significantly increased DPC proliferation relative to untreated controls (Fig 1). The highest degree of proliferation was achieved using 20 μM oleuropein. The effect of 20 μM oleuropein on increasing cell proliferation was significantly higher than that of minoxidil at 100 μM.

### Oleuropein upregulates the Wnt/β-catenin pathway in DPCs

**Fig 2:**
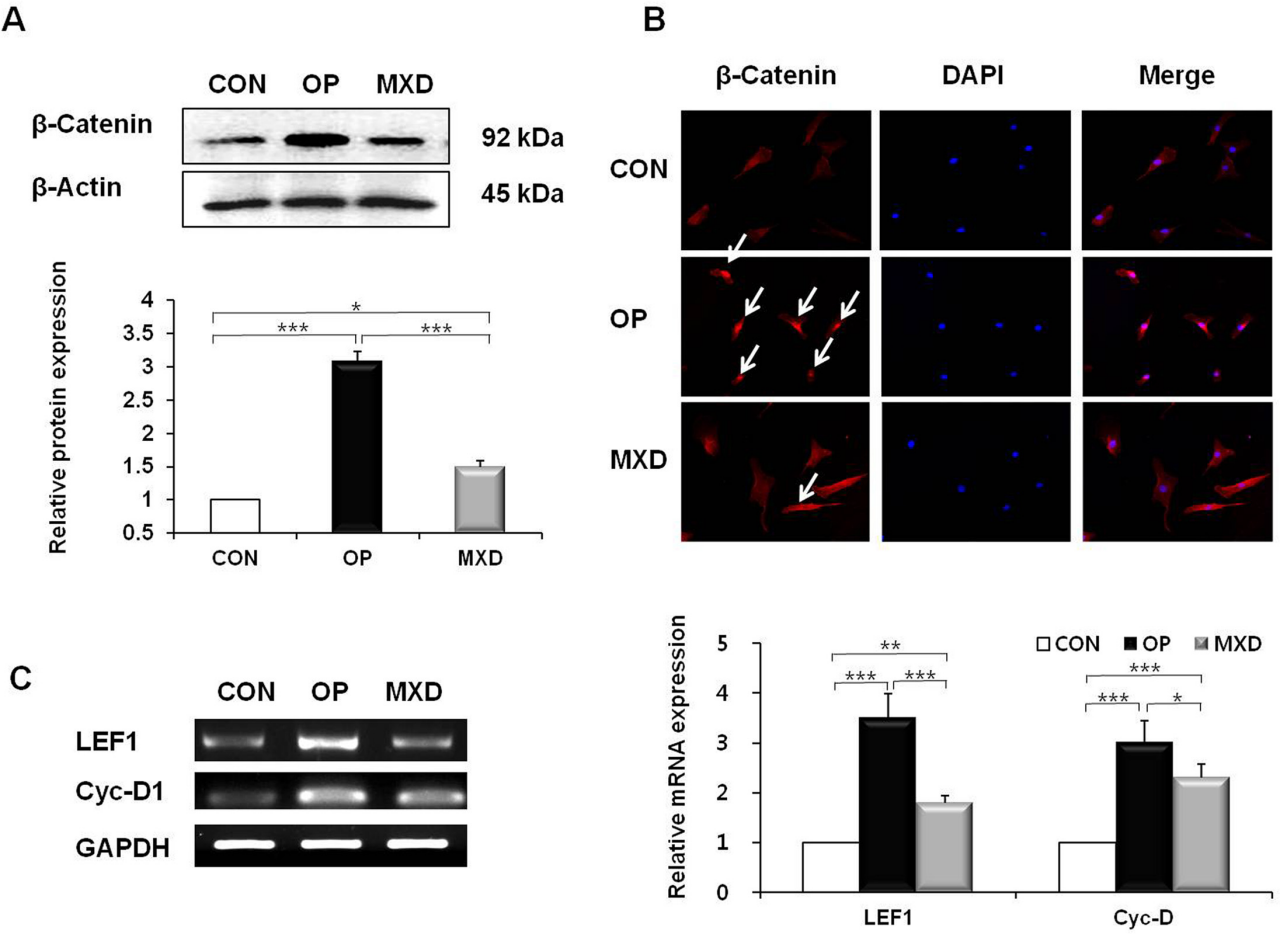
Effect of oleuropein on the activation status of the Wnt/β-catenin pathway in DPCs. DPCs were treated with 20 μM OP or 100 μM MXD for 24 h. (A) The protein level of β-catenin in the DPCs was determined by western blotting. A representative image of triplicate experiments is shown in the upper panel. The lower panel shows the intensity of the bands that were densitometrically measured and normalized against the protein level of β-actin. (B) DPCs were immunocytochemically stained with β-catenin antibody (left column panel), and the corresponding DAPI nuclear staining is also shown (middle column panel); merged images are shown in the right column panel. Original magnification: 20×. (C) LEF1 and Cyc-D1 mRNA levels were measured by RT-PCR. A representative image of triplicate experiments is shown in the left panel. The right panel shows the intensity of the bands that were densitometrically measured and normalized against the mRNA expression level of GAPDH. The western blotting and RT-PCR results are the mean ± SD from duplicates of three independent experiments. * = *p* < 0.05, ** = *p* < 0.01, *** = *p* < 0.001 (one-way ANOVA, Tukey's test).

To investigate whether oleuropein modulates the Wnt/β-catenin pathway in DPCs, we examined the expression of nuclear β-catenin by western blotting analysis. As shown in Fig 2A, the protein level of nuclear β-catenin was significantly increased in oleuropein-treated DPCs (a 212% increase) compared with control cells treated with dimethyl sulfoxide (DMSO). Next, we performed immunocytochemistry to study the localization of β-catenin in DPCs after treating the cells with oleuropein for 24 h. Cells treated with oleuropein showed significant accumulation of β-catenin in the nucleus compared with the DMSO control (Fig 2B). Cells treated with oleuropein for 24 h were evaluated for expression of Wnt/β-catenin signaling target genes by using RT-PCR. We found that compared with control cells treated with DMSO, cells treated with oleuropein showed significantly increased expression of LEF1 (*P* = 0.0027) and Cyc-D1 (*P* = 0.0034) (Fig 2C). In addition, cells treated with oleuropein showed significantly increased nuclear accumulation of β-catenin and mRNA expression of LEF1 and Cyc-D1 relative to those treated with minoxidil.

### Effect of oleuropein on anagen-phase induction in C57BL/6N mice

**Fig 3:**
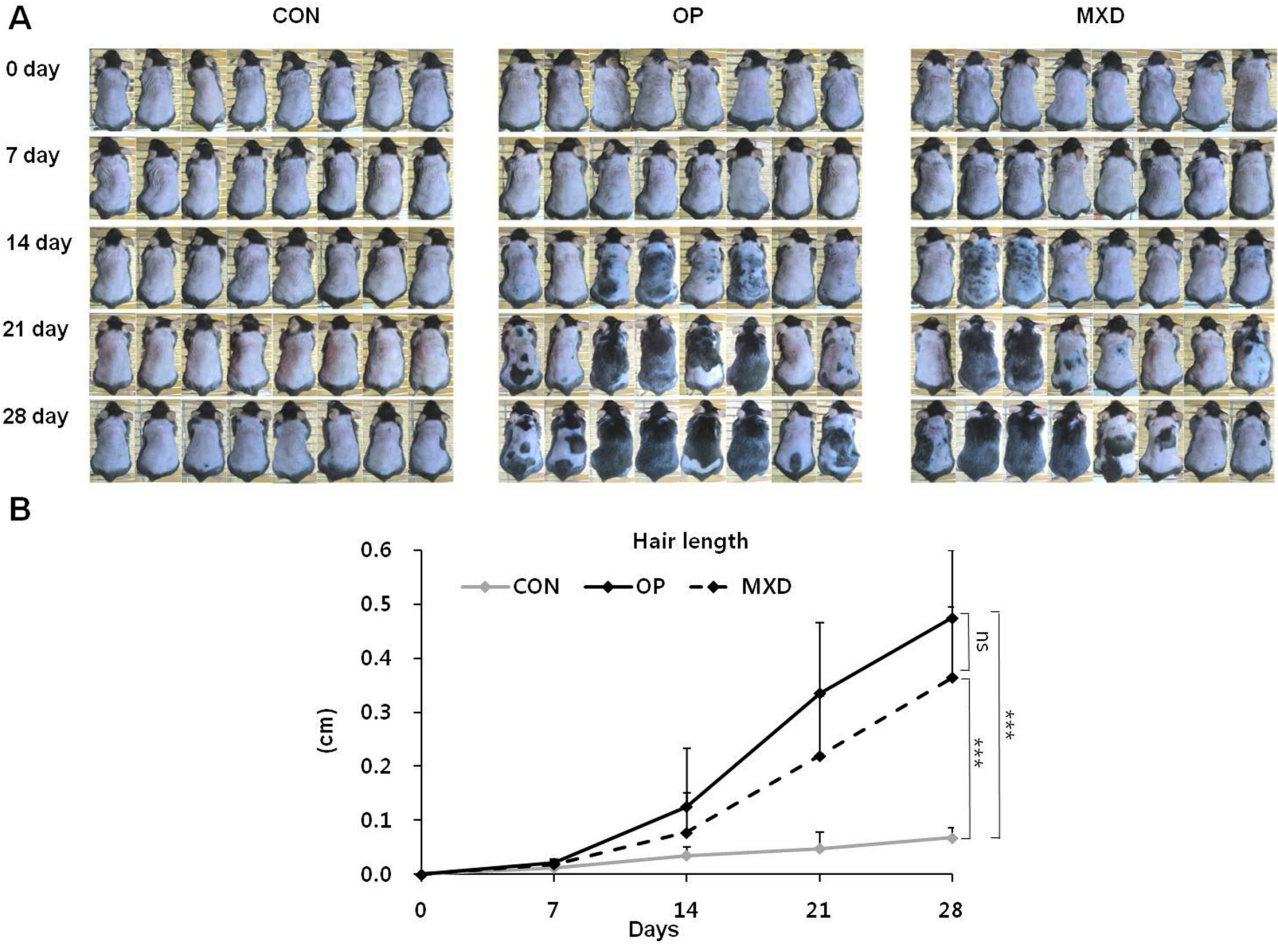
Hair growth-promoting effect of oleuropein in C57BL/6N mice. The back skin of male C57BL/6N mice (eight weeks old) was shaved and treated daily with CON, 0.4 mg OP, or 3 mg MXD for 28 days. (A) The back skin was photographed at 0, 7, 14, 21, and 28 days. (B) The length of randomly plucked hairs was measured at different time intervals (0, 7, 14, 21, and 28 days) after topical application of CON, OP, or MXD. Values are the mean ± SD of eight mice. ns = *p* > 0.05, *** = *p* < 0.001 (one-way ANOVA, Tukey's test).

We used C57BL/6N mice to investigate whether anagen-phase induction was promoted by oleuropein. C57BL/6N mouse dorsal hair is known to have a time-synchronized hair growth cycle. As shown in Fig 3A, oleuropein- and minoxidil-treated groups exhibited gray skin at day 14 after hair-growth induction, and their hair shafts were visible at day 14. The skin color of the control group remained less pigmented until day 14. The hair shafts of this group were not visible until day 21. At day 28, the area of black skin was significantly larger in the oleuropein-treated group than that in the control group. The minoxidil-treated group showed a smaller area of black skin than the oleuropein-treated group. To determine whether oleuropein promoted hair growth, we measured the length of 10 hairs plucked from the dorsal skin center area of each mouse at 0, 7, 14, 21, and 28 days. As shown in Fig 3B, the hairs in the oleuropein-treated group were significantly longer than those in the control group. Moreover, the hairs in the oleuropein-treated group were observed to be longer than those in the minoxidil-treated group.

### Effects of oleuropein on the structure of mouse hair follicles

**Fig 4:**
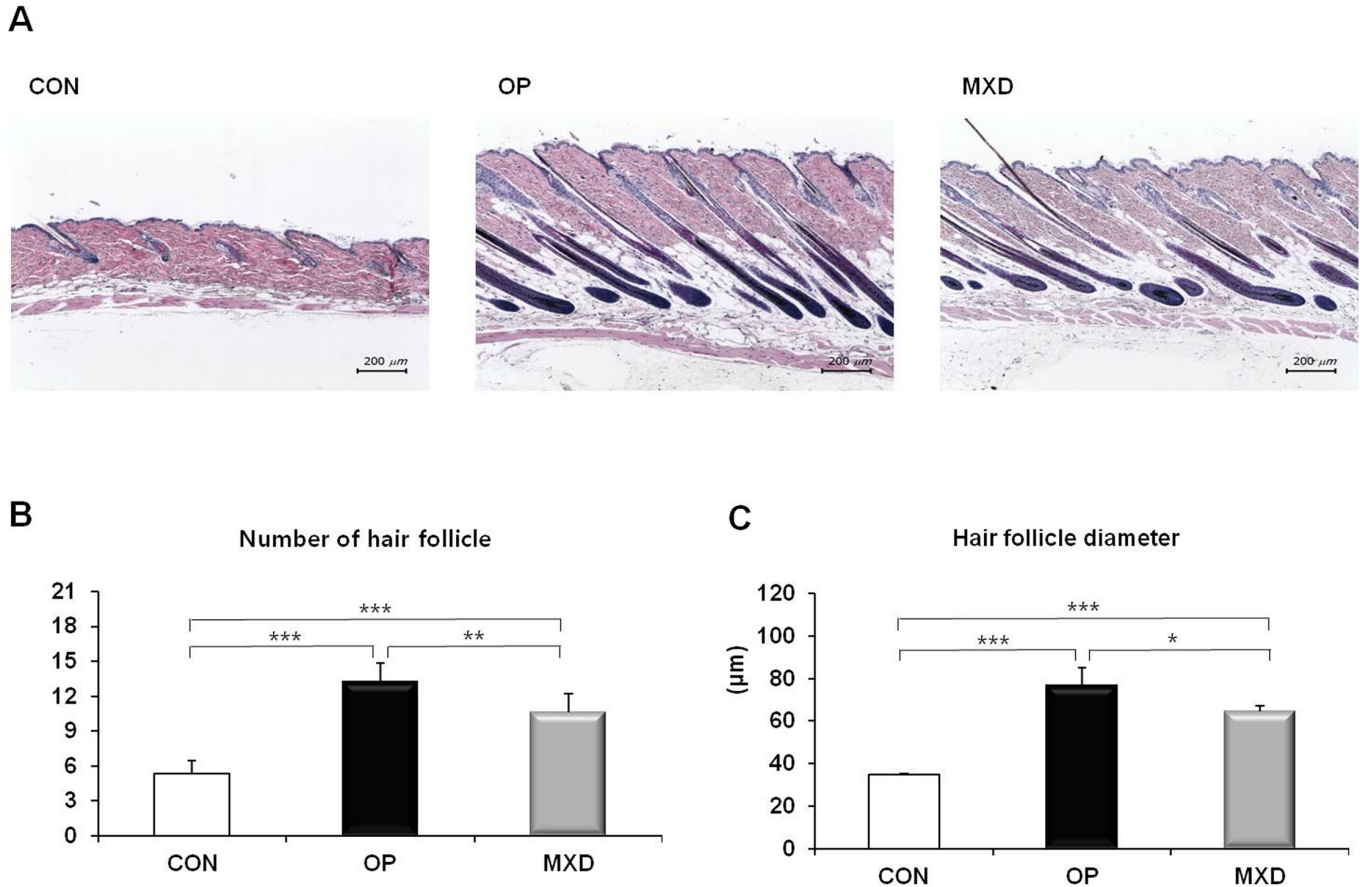
Effect of oleuropein on hair follicle growth in C57BL/6N mice. The CON, 0.4 mg/mL OP, or 3 mg/mL MXD was topically applied to the shaved back skin of C57BL/6N mice for 28 days. (A) The effect of oleuropein on the hair follicles of the mice was analyzed by using hematoxylin and eosin staining. Longitudinal sections of the back skin were stained, and representative photomicrographs of skin sections are shown. Bars, 200 μm. Number (B) and diameter (C) of hair follicles in deep subcutis. Values are the mean ± SD of eight mice. * = *p* < 0.05, ** = *p* < 0.01, *** = *p* < 0.001 (one-way ANOVA, Tukey's test).

An increase in the number and size of hair follicles is thought to indicate the transition of hair growth from the telogen to anagen phase [14]. To investigate the progression of hair follicles in the hair cycle, we performed H&E staining of skin biopsy sections. In the representative longitudinal sections, the number and size of hair follicles were significantly increased in the oleuropein-treated group compared to the control group (Fig 4). In addition, the number and size of hair follicles in the oleuropein-treated group were significantly greater than those of minoxidil-treated group.

### Effects of oleuropein on growth factor expression in mice

**Fig 5:**
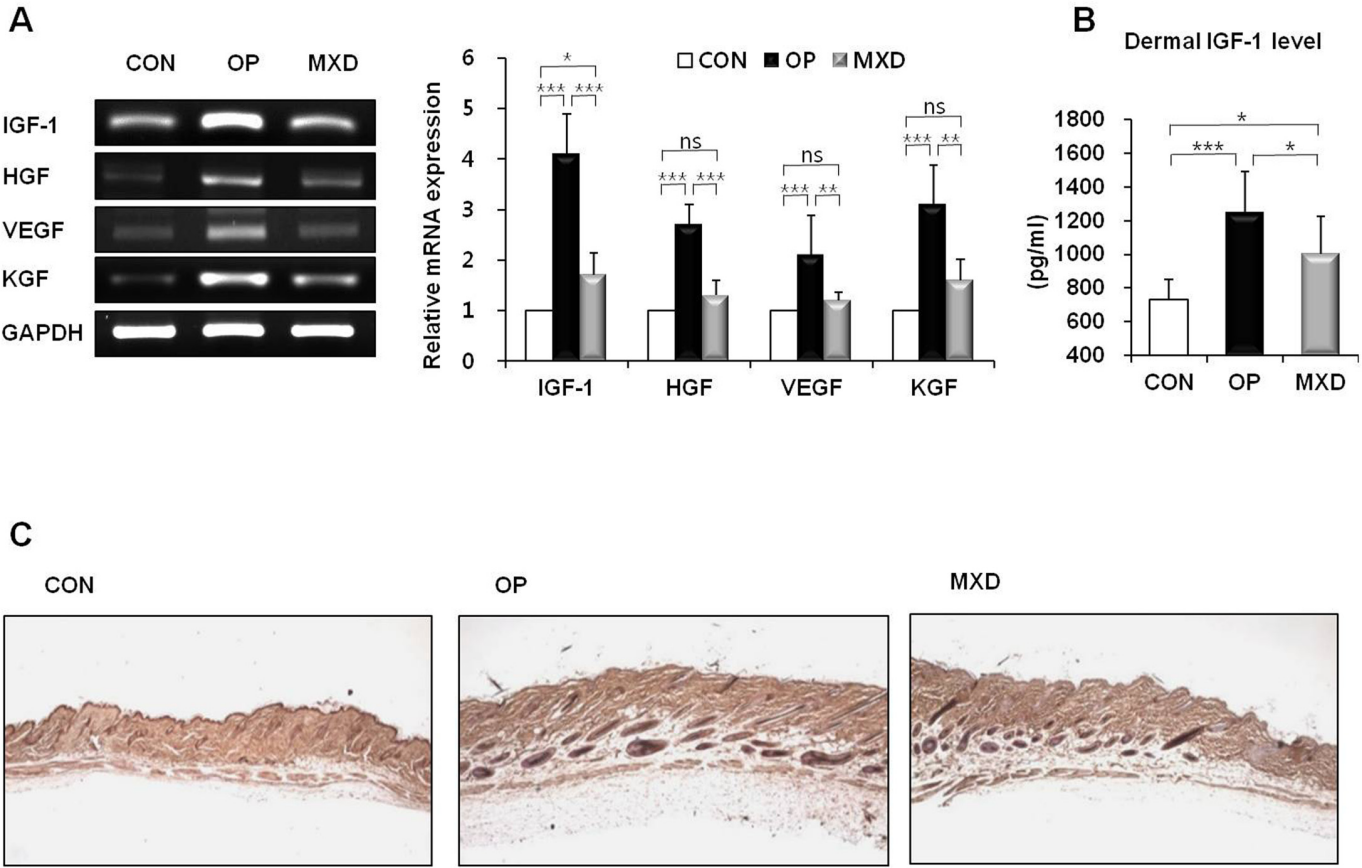
Effect of oleuropein on mRNA and protein expression of growth factors in C57BL/6N mouse skin tissue. (A) The IGF-1, HGF, VEGF, and KGF mRNA expression levels were measured by RT-PCR. A representative image of triplicate experiments is shown in the left panel. The right panel shows the intensity of the bands that were densitometrically measured and normalized against the mRNA expression level of GAPDH. (B) Dermal levels of IGF-1 were assessed by ELISA. (C) Immunohistochemical analysis of IGF-1 expression in hair follicle. Original magnification: 40×. The RT-PCR results are the mean (*n* = 8) ± SD of three independent experiments (*n* = 2 or 3 per experiment) for each group. ns = *p* > 0.05, * = *p* < 0.05, ** = *p* < 0.01, *** = *p* < 0.001 (one-way ANOVA, Tukey's test).

Mice treated with oleuropein exhibited a significant increase in the mRNA levels of IGF-1 (*P* = 0.0008), hepatocyte growth factor (HGF, *P* = 0.0021), vascular endothelial growth factor (VEGF, *P* = 0.0062), and keratocyte growth factor (KGF, *P* = 0.0052) in their skin tissues compared with mice treated with vehicle (Fig 5A). The effect of oleuropein on increasing mRNA levels of these growth factors was significantly greater than that of minoxidil. ELISA analysis revealed that dermal IGF-1 levels were significantly increased in mice treated with oleuropein (71% increase) compared with mice treated with vehicle only (Fig 5B). Consistent with these results, IGF-1 expression in hair follicles detected using immunohistochemistry was increased after oleuropein treatment compared with the control vehicle (Fig 5C). The oleuropein-treated mice demonstrated significant increases in dermal IGF-1 level and immunohistochemical expression compared with control mice (Fig 5B and 5C).

### Oleuropein modulates the Wnt/β-catenin pathway in mice

**Fig 6:**
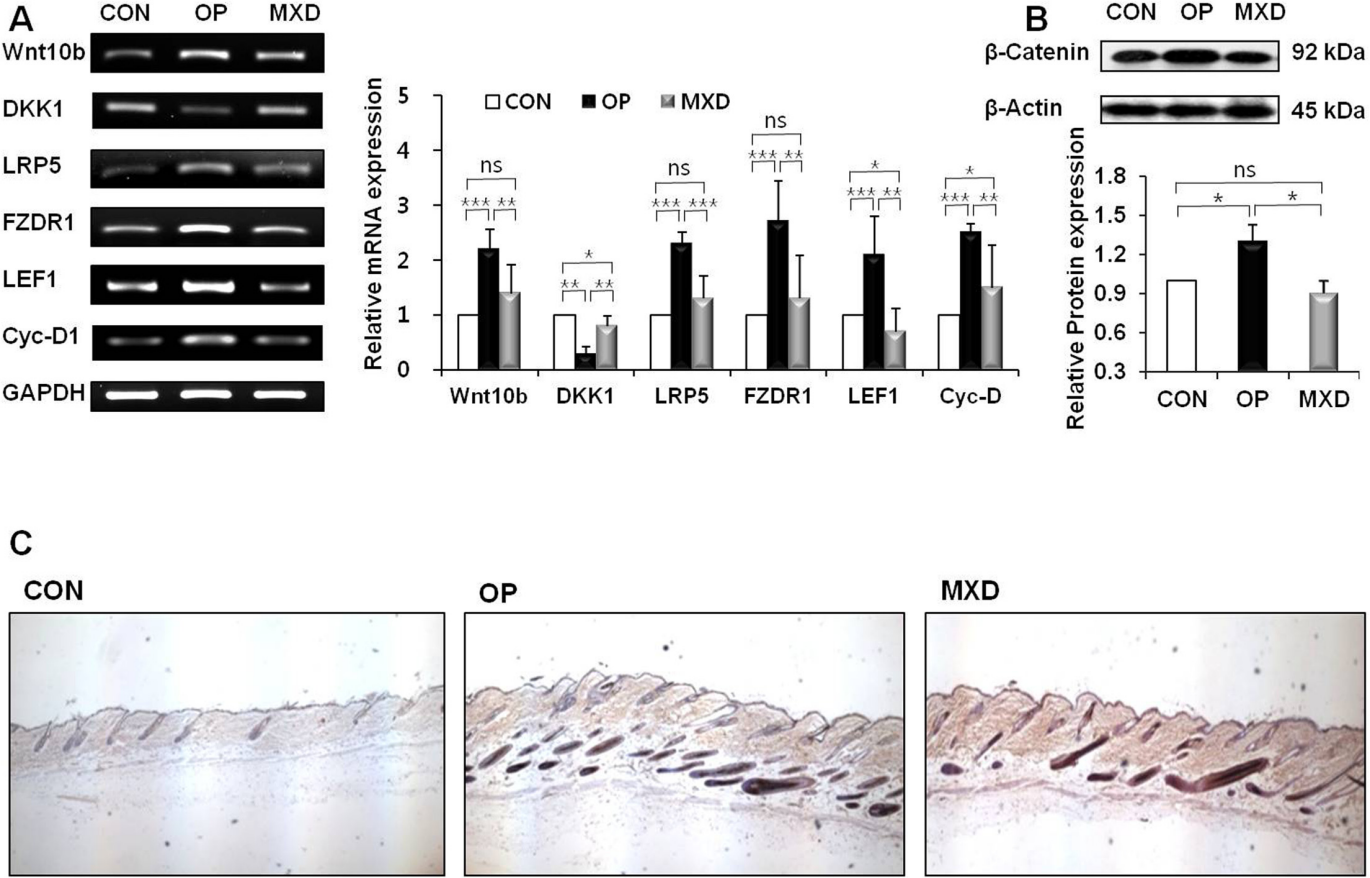
Effect of oleuropein on expression of genes related to hair growth in C57BL/6N mice skin tissue. (A) The Wnt10b, DKK1, LRP5, FZDR1, LEF1, and Cyc-D1 mRNA expression levels were measured by RT-PCR. A representative image of triplicate experiments is shown in the left panel. The right panel shows the intensity of the bands that were densitometrically measured and normalized against the mRNA expression level of GAPDH. (B) The protein level of β-catenin in C57BL/6N mouse skin tissue was determined by western blotting. The lower panel shows the intensity of the bands that were densitometrically measured and normalized against the protein level of β-actin. (C) Immunohistochemical analysis of β-catenin in hair follicles. Original magnification: 40×. Western blotting and RT-PCR results are the mean (*n* = 8) ± SD of three independent experiments (*n* = 2 or 3 per experiment) for each group. ns = *p* > 0.05, * = *p* < 0.05, ** = *p* < 0.01, *** = *p* < 0.001 (one-way ANOVA, Tukey's test).

Mice treated with oleuropein exhibited a significant increase in the mRNA levels of WNT10b (*P* = 0.0045), LRP5 (*P* = 0.0004), FZDR1 (*P* = 0.0123), and the Wnt-responsive transcription factor LEF1 (*P* = 0.0142), along with its target genes, such as Cyc-D1 (*P* = 0.009), in their skin tissues compared with mice treated with the control vehicle (Fig 6A). In addition, oleuropein-treated mice showed a significant decrease in the mRNA level of DKK1 (*P* = 0.0003) in their skin tissues compared with control vehicle-treated mice. The oleuropein-treated mice showed significant increases in the mRNA levels of Wnt signaling-related genes (Wnt10b, LRP5, FZDR1, LEF1, and Cyc-D1) relative to minoxidil-treated mice. Western blot analysis of the skin tissue of mice revealed a significantly increased protein level of β-catenin (27% increase) in oleuropein-treated mice relative to control group animals (Fig 6B). Consistent with this result, β-catenin expression in hair follicles detected using immunohistochemistry was increased after oleuropein treatment compared with the control (Fig 6C). This effect of oleuropein on increasing protein levels of β-catenin in hair follicles was greater than that of minoxidil.

### Direct or indirect regulation of genes by oleuropein

**Fig 7:**
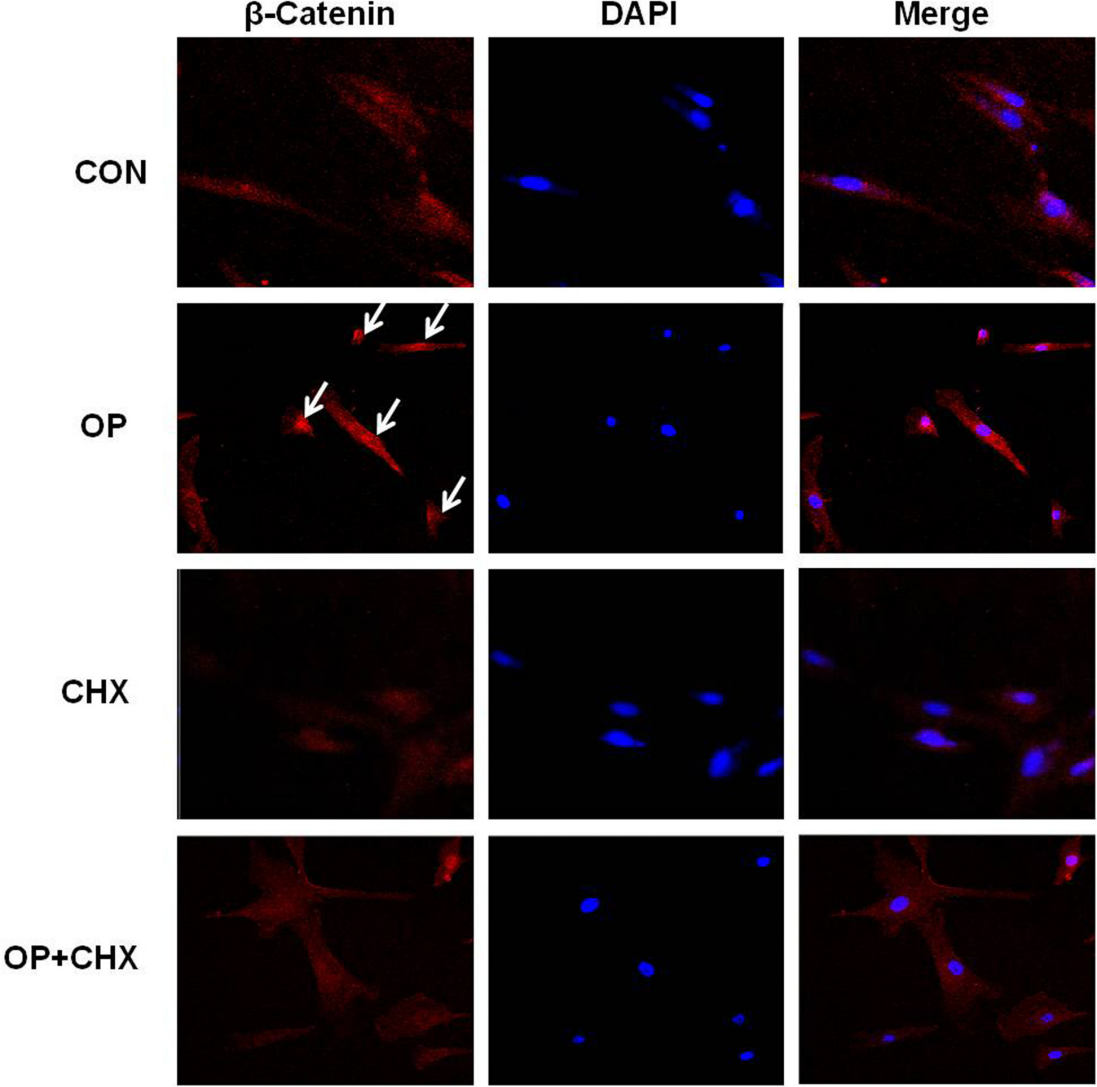
Effects of cycloheximide on β-catenin accumulation in the nucleus of DPCs induced by oleuropein treatment. DPCs were allowed to attach overnight and then treated (60 min) with cycloheximide (10 μg/ml) and thereafter switched to medium with or without oleuroepin (20 μM) in the presence of cycloheximide (10 μg/ml) for 24 h. Then cells were stained for β-catenin immunofluorescence (red) and counterstained with DAPI (blue). Merged image of β-catenin-rhodamine and DAPI staining is also shown. Original magnification: 20×.

**Fig 8:**
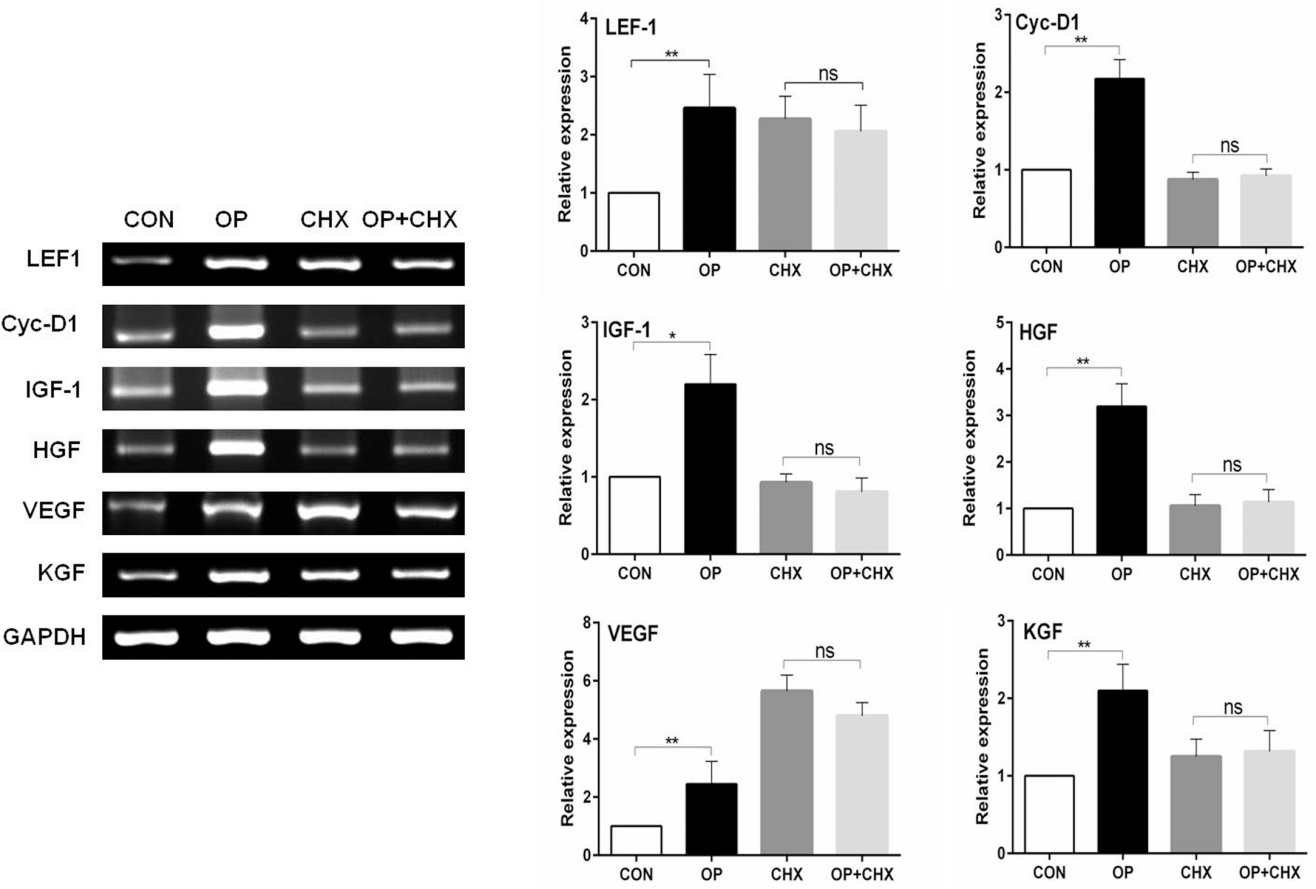
Effects of cycloheximide on gene expression changes in DPCs induced by oleuropein treatment. DPCs were allowed to attach overnight and then treated (60 min) with cycloheximide (10 μg/ml) and thereafter switched to medium with or without oleuroepin (20 μM) in the presence of cycloheximide (10 μg/ml) for 24 h. mRNA expression levels of LEF1, Cyc-D1, IGF-1, HGF, VEGF, and KGF were measured by RT-PCR. A representative image of triplicate experiments is shown in the left panel. The right panel shows the intensity of the bands that were densitometrically measured and normalized against the mRNA expression level of GAPDH. The RT-PCR results are the mean ± SD from duplicates of three independent experiments. ns = *P* > 0.05, * = *p* < 0.05, ** = *p* < 0.01 (student’s t-test).

We described here oleuroepin-induced stimulation of the Wnt/β-catenin signaling pathway both *in vitro* and *in vivo* and the upregulation of IGF-1, KGF, HGF, VEGF gene expression in mouse skin tissues. To check whether the effects of oleuroepin were due to a direct action on β-catenin accumulation and gene transcription or mediated through increased expression of oleuroepin-dependent auxiliary proteins, we analyzed the effect of oleuropein in the presence of the protein synthesis inhibitor, CHX (Fig 7). Our results showed that the accumulation of β-catenin by oleuropein, as determined by immunocytochemistry, was blocked by cycloheximide (Fig 7). The expressions of Wnt target genes, such as LEF1 and Cyc-D1, and several growth factors, including IGF-1, HGF, VEGF, and KGF, were upregulated by oleuropein within 24 h, but the effect of oleuropein was indirect since induction on these genes was abolished by CHX (Fig 8).

## Discussion

In the present study, oleuropein was topically applied to the shaved dorsal skin of telogenic C57BL/6N mice. The induction of anagenic hair growth in telogenic mouse skin by a substance may depend on the method of administration. Yamamoto *et al*. reported that topical application of FK506, a potent immunosuppressant, to the dorsal skin of CD-1 mice stimulated hair growth in a dose-dependent manner, whereas oral administration of FK506 did not stimulate significant hair growth [15]. Recently, Jo *et al*. also reported that in an animal study using C57BL/6 female mice, oral administration of valproic acid failed to promote hair growth, whereas topical valproic acid application significantly promoted hair growth [16]. Oleuropein is expected to be easily absorbed with topical application in the skin because of its relatively low molecular weight (540 g/mol). Peurta *et al*. have reported that topical application of oleuropein at a dose of 1.0 mg per mouse significantly reduced edema in an arachidonic acid-induced inflammation model [17]. Based on our preliminary study involving different oleuropein dosages (0.4 to 3.0 mg/mous), 0.4 mg of oleuropein per mouse was found to be the minimal effective dose for anagen hair induction in C57BL/6N mice.

In the present study, mice topically treated with oleuropein did not develop evident adverse effects. In acute toxicity studies for oleuropein, no lethality or adverse effects were observed in mice even when it was administered at doses as high as 1,000 mg/kg; therefore, an LD_50_ value could not be determined [18]. A commercially available olive leaf extract EFLA943 was standardized to an oleuropein content of 18–26% and studied to evaluate its safety and anti-hypertensive effects [19]. In a randomized, parallel, double-blind and active-controlled clinical study conducted on 89 adults with stage-1 hypertension in Indonesia, EFLA943 treatment at a dose of 1,000 mg (containing 180–260 mg of oleuropein) daily for 8 weeks did not affect hepatic and renal function [19], and it also did not affect the hematological parameters and electrolyte balance of study participants [19].

Mammalian hair follicles are composed of two type of cells, dermal papilla and epithelial cells [20]. Generally, DPCs are considered as inducers and epithelial cells as responders in the process of hair formation, although the signaling between the two cell types is reciprocal and complicated [21]. DPCs are known to be responsible for the induction and maintenance of the growth and differentiation of epithelial cells [22,23]. They instruct the surrounding epithelial cells to proliferate, move upward, and differentiate into the hair shaft and the channel surrounding the hair shaft, called the inner root sheath [1,3]. The size of individual hairs is probably correlated with the number of DPCs [24,25]. Thus, the proliferative potential of DPCs is thought to be one of important parameters that regulate hair growth. In this regard, oleuropein could promote hair growth by inducing proliferation of DPCs.

The basis for hair follicle regeneration relies on the unique follicular epithelial and mesenchymal components (dermal papilla) and their interactions [26]. The inductive signals exchanged between follicular dermal papilla and epithelial cells are largely unknown; however, several studies have suggested that Wnt/β-catenin signaling pathways may be involved [27,28]. Upon activation of the Wnt/β-catenin pathway, β-catenin accumulates in the cytoplasm and is transported to the nucleus, where it interacts with members of the TCF/LEF family of transcription factors to activate target gene expression [29,30]. Li *et al*. reported that Wnt10b could induce the biological switch of hair follicles from the telogenic phase to the anagenic phase in mouse skin [31]. In addition, they showed that siRNA suppression of β-catenin inhibited hair follicle regeneration, even when Wnt10b was overexpressed [31]. Recently, Choi *et al*. reported that β-catenin-deleted and DKK1-expressing hair follicles showed greatly diminished expression of Cyc-D1, a direct Wnt/β-catenin target gene that helps initiate the transition from late G1 to S phase of the cell cycle, likely contributing to decreased hair follicle matrix proliferation [32]. In the present study, in parallel to the significant upregulation of Wnt10b, LRP5, FZDR1, β-catenin, LEF1, and Cyc-D1, a prominent decrease of DKK1 levels was observed in skin tissue of oleuropein-treated mice compared with the control group. On the basis of these results, we propose that oleuropein may cause premature entry of telogenic follicles into the anagen phase via activation of the Wnt10b/β-catenin signaling pathway.

Recent studies suggested that besides inducing telogen-to-anagen transition, Wnt/β-catenin signaling has a role in maintaining anagen [6, 33]. Deletion of the β-catenin gene in DPCs of fully developed hair follicles caused premature induction of the catagen phase of the hair cycle in mice, indicating that activation of β-catenin in DPCs can prolong the anagen phase as well [6]. In line with this, Kwack *et al*. observed that DKK-1, a potent antagonist of the Wnt/β-catenin signaling pathway, promoted catagen progression, whereas neutralizing antibody against DKK-1 delayed catagen progression, thereby prolonging anagen [33].

In addition to Wnt, several different growth factors such as IGF-1, KGF, and HGF have been reported to influence the growth and development of hair follicles [34–37]. IGF-1 shares considerable structural homology with insulin and exerts insulin-like effects on food intake and glucose metabolism. It has also been suggested to play a role in regulating cellular proliferation during the development of hair follicles [34,37]. KGF has been identified as a paracrine mediator of proliferation in a wide variety of epithelial cells including hepatocytes and gastrointestinal epithelial cells [38]. In the skin, KGF was found to stimulate the proliferation and differentiation of epithelial cells within hair follicles [35]. HGF was initially characterized as a powerful mitogen for hepatocytes and a stimulator of epithelial cell dissociation [39]. Recently, it has been reported that HGF has morphogenic activity for epithelial cells during the anagen phase [36]. IGF-1, KGF, and HGF exert their effects by binding to specific, high-affinity receptor molecules (IGF-1Rs, KGFRs, and C-METs) on the cell surface. Activation of the receptors ultimately results in the activation of at least two main signaling pathways: the Ras/Raf/mitogen-activated protein kinase (MAPK) pathway and the phosphoinositide-3 kinase (PI3K)/AKT pathway [40–42]. The MAPK and AKT pathways regulate levels of cell cycle proteins such as Cyc-D1 to increase cell proliferation [43]. In the present study, oleuropein increased IGF-1, KGF, and HGF mRNA levels in mouse skin tissue, which suggests that oleuropein may produce positive effects on hair growth partly by regulating the gene expression of IGF-1, KGF, and HGF in skin tissue.

In addition to hair follicle tissue remodeling, skin vascular networks also undergo substantial changes with the progression of the anagen stage [44]. Cyclic hair growth is dependent on the induction of angiogenesis to meet the increased nutritional needs of hair follicles during the anagen phase of rapid cell division [45]. Perifollicular angiogenesis temporally and spatially correlates with upregulation of VEGF, a potent angiogenic growth factor [45]. VEGF has been shown to promote angiogenesis through activation of several different intracellular signaling pathways, including the MAPK and PI3K/AKT pathways [46]. Our *in vivo* findings demonstrate that oleuropein significantly stimulated the expression of VEGF in mouse skin tissue. This finding suggests that oleuropein could promote the induction of angiogenesis during the anagen phase by regulating the expression of VEGF in skin tissue, thereby promoting hair growth.

In summary, our present findings indicate that topical oleuropein administration at a dose of 0.4 mg per mouse induces anagenic hair growth in telogenic C57BL/6N mouse skin. The stimulation of the Wnt10b/β-catenin signaling pathway and the upregulation of IGF-1, KGF, HGF, and VEGF gene expression in mouse skin tissue may be of high relevance and contributing to the hair growth-promoting effect of oleuropein. Even though mice are commonly used as skin disease models, there are inherent differences in skin structure between mice and human skin. For example, the hair follicle density in mice is much greater than that in humans making the resulting interfollicular region of skin thinner, potentially increasing the rate of chemical absorption [47]. Additional research is required to further determine the potential for developing oleuropein as a pharmacologic strategy for promoting hair growth.
